# Supplementary material for: Generative AI enables medical image segmentation in ultra low-data regimes
Source: Nat Commun. 2025 Jul 14;16:6486. doi: 10.1038/s41467-025-61754-6 (PMC12260076; doi:10.1038/s41467-025-61754-6)
Supplement: Supplementary file 1 — Supplementary Information [file 41467_2025_61754_MOESM1_ESM.pdf]

Supplementary Information for "Generative AI Enables Medical Image Segmentation in Ultra Low-Data Regimes"

Li Zhang[1], Basu Jindal[1], Ahmed Alaa[2,3], Robert Weinreb[4], David Wilson[5], Eran Segal[6,7], James Zou[8,9], and Pengtao Xie[1,10] ✉

[1]Department of Electrical and Computer Engineering, University of California San Diego, La Jolla, CA, 92093, USA
[2]Bakar Computational Health Sciences Institute, University of California San Francisco, San Francisco, CA, 94143, USA
[3]Department of Electrical Engineering and Computer Sciences, University of California Berkeley, Berkeley, CA, 94720, USA
[4]Hamilton Glaucoma Center, Shiley Eye Institute, Viterbi Family Department of Ophthalmology, University of California San Diego, La Jolla, CA, 92093, USA
[5]Division of Pulmonary, Allergy and Critical Care Medicine, Department of Medicine, University of Pittsburgh, Pittsburgh, PA, 15261, USA
[6]Department of Computer Science and Applied Mathematics, Weizmann Institute of Science, Rehovot, 7610001, Israel
[7]Department of Molecular Cell Biology, Weizmann Institute of Science, Rehovot, 7610001, Israel
[8]Department of Biomedical Data Science, Stanford University School of Medicine, Stanford, CA, 94305, USA
[9]Department of Computer Science, Stanford University, Stanford, CA, 94305, USA
[10]Department of Medicine, University of California San Diego, La Jolla, CA, 92093, USA

Correspondence: ***p1xie@ucsd.edu***

Task	Dataset	Train	Validate	Test
Skin lesion segmentation	ISIC	160	40	594
	PH2	-	-	200
	DermIS	-	-	98
	DermQuest	32	8	61
Lung segmentation	JSRT	140	35	72
	NLM-MC	-	-	138
	NLM-SZ	-	-	566
	COVID	8	2	583
Breast cancer segmentation	BUID	80	20	230
Placental vessel segmentation	FPD	80	20	182
	FetReg	80	20	658
Polyp segmentation	KVASIR	480	120	200
	CVC-Clinic	80	20	212
Foot ulcer segmentation	FUSeg	480	120	200
Intraretinal cystoid segmentation	ICFluid	40	10	460
Left ventricle segmentation	ETAB (Left ventricle)	8	2	50
Myocardial wall segmentation	ETAB (Myocardial wall)	8	2	50
Hippocampus segmentation	MSD-Hippocampus	166	42	52
Liver segmentation	MSD-Liver	78	20	25

Supplementary Table 1 | Dataset statistics. Detailed splits for datasets used for model training, validation, and testing across all medical image segmentation tasks. Each row specifies the dataset associated with a given segmentation task, along with the number of images (or cases) used for training, validation, and testing.

**Method**	**Dataset**	**P-value**	**Effect size**	**T-statistic**
Learnable Multi-branch vs Single-branch	ISIC-200	0.016	3.462	4.239
	ICFluid-50	0.007	6.566	8.042
	BUID-100	0.001	8.121	9.946
Fixed Multi-branch vs Single-branch	ISIC-200	0.128	1.603	1.964
	ICFluid-50	0.002	7.401	9.065
	BUID-100	0.038	2.745	3.362
Learnable Multi-branch vs Fixed Multi-branch	ISIC-200	0.051	2.249	2.755
	ICFluid-50	0.037	3.709	4.544
	BUID-100	0.032	3.021	3.699

Supplementary Table 2 | Statistical comparison of segmentation performance across models and datasets. Pairwise comparisons were conducted using two-sided paired t-tests on performance scores across three independent training runs (random seed replicates) for each method. Each test compares the segmentation performance of two models (as indicated in the "Method" column) on a specific dataset. Reported values include exact P-values, Cohen's d effect sizes, and t-statistics with 2 degrees of freedom (df = 2). No adjustments for multiple comparisons were applied.

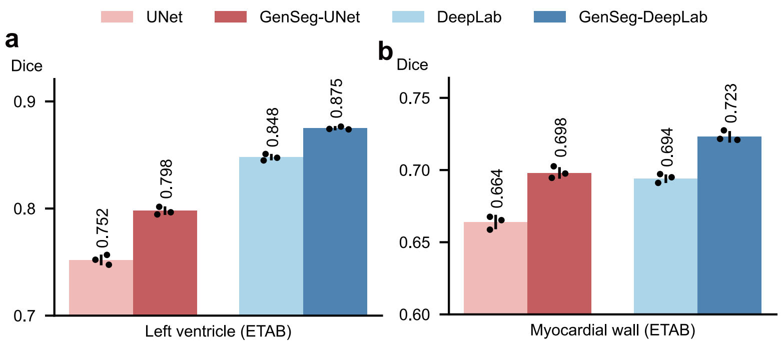

Supplementary Fig. 1 | Performance of GenSeg on the ETAB benchmark. The performance of GenSeg applied to UNet (GenSeg-UNet) and DeepLab (GenSeg-DeepLab) under in-domain settings (test and training data are from the same domain) in the tasks of segmenting left ventricles (**a**) and myocardial wall (**b**) using 8 training examples from the ETAB dataset, compared to vanilla UNet and DeepLab. In all panels, bar heights represent the mean, and error bars indicate the standard deviation across three independent runs with different random seeds. Results from individual runs are shown as dot points. Source data are provided as a Source Data file.

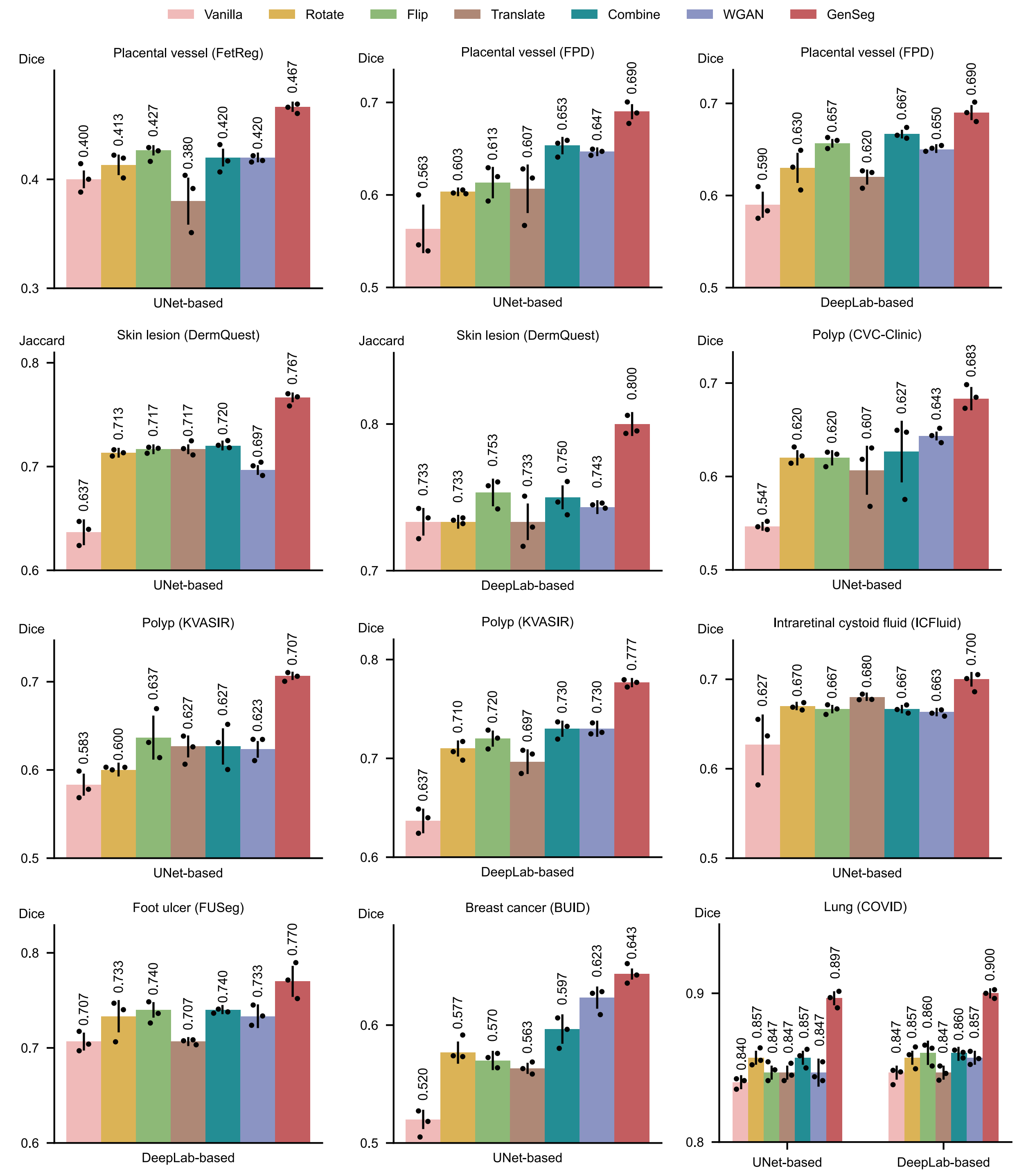

Supplementary Fig. 2 | Further comparison of GegSeg with data augmentation and generation methods. GenSeg's in-domain generalization performance compared to baseline methods including Rotate, Flip, Translate, Combine, and WGAN, when used with UNet or DeepLab in segmenting placental vessels, skin lesions, polyps, intraretinal cystoid fluids, foot ulcers, breast cancer, and lungs, using the FetReg, FPD, DermQuest, CVC-Clinic, KVASIR, ICFluid, FUSeg, BUID, and COVID datasets. In all panels, bar heights represent the mean, and error bars indicate the standard deviation across three independent runs with different random seeds. Results from individual runs are shown as dot points. Source data are provided as a Source Data file.

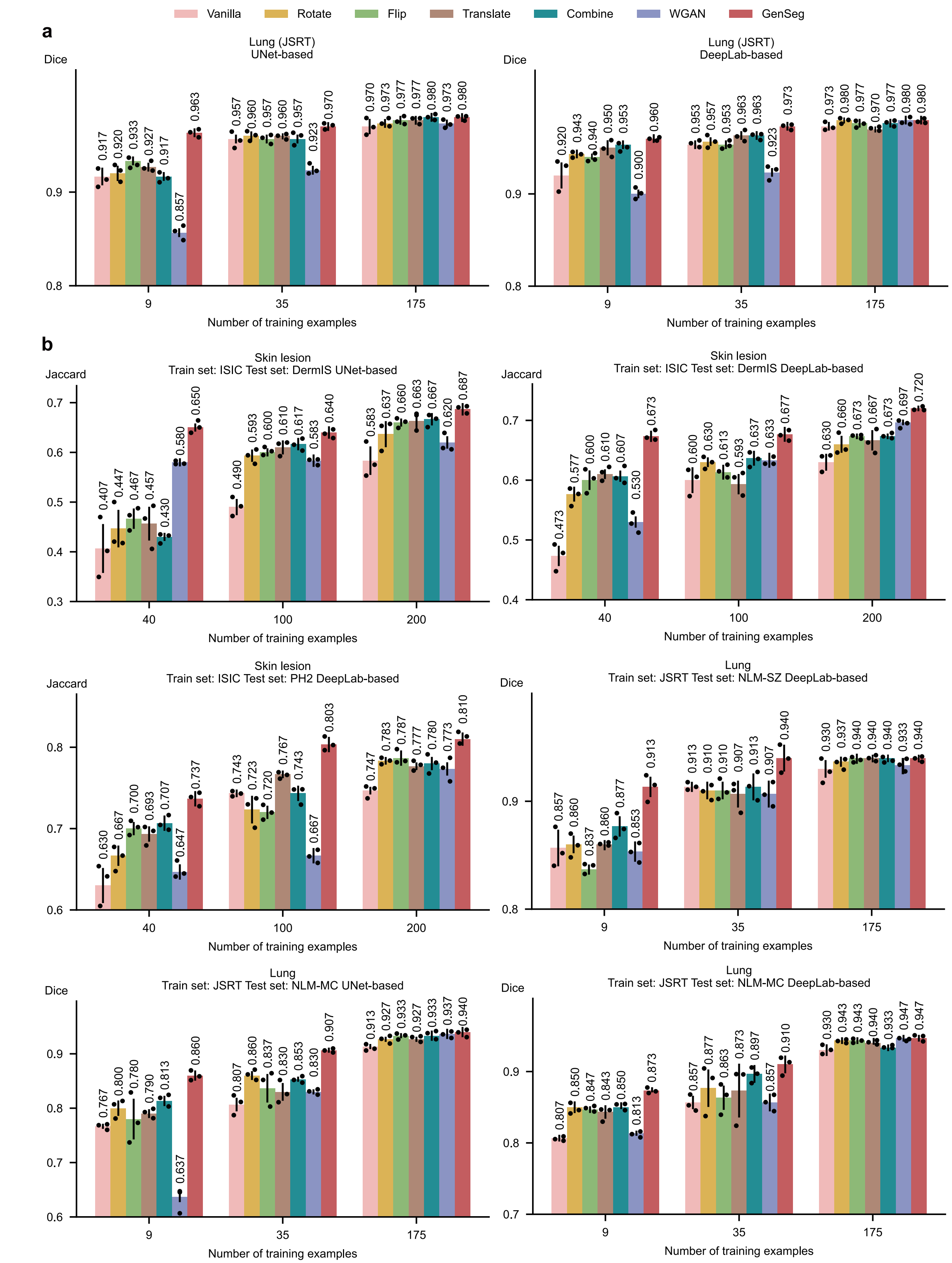

Supplementary Fig. 3 | Further comparison of GegSeg with data augmentation and generation methods across varying numbers of training examples. a, Comparison of in-domain generalization performance for lung segmentation using the JSRT dataset. **b**, Comparison of out-of-domain generalization performance in segmenting skin lesions (using the ISIC dataset for training, DermIS and PH2 for testing) and lungs (using JSRT for training, NLM-SZ and NLM-MC for testing). In all panels, bar heights represent the mean, and error bars indicate the standard deviation across three independent runs with different random seeds. Results from individual runs are shown as dot points. Source data are provided as a Source Data file.

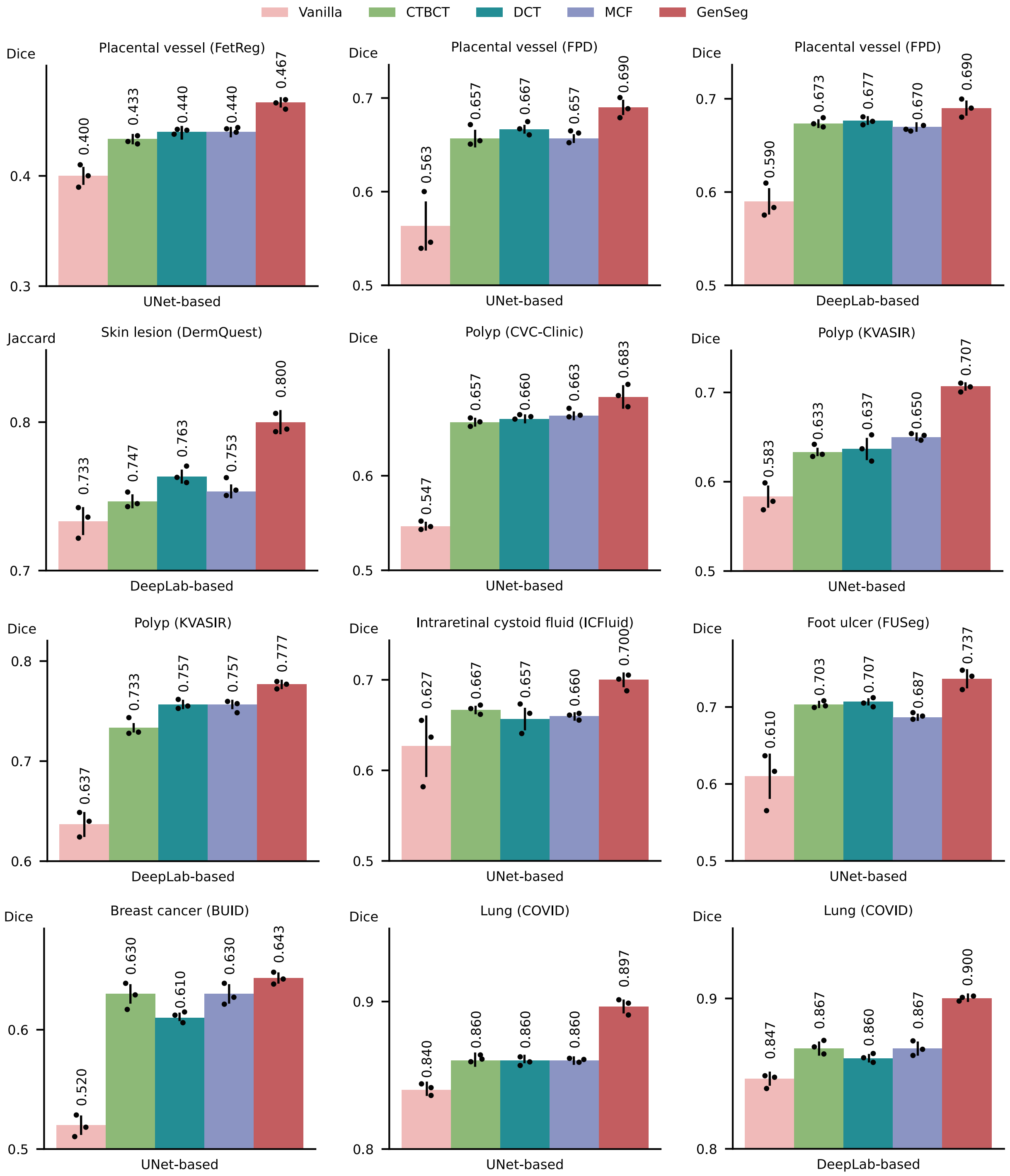

Supplementary Fig. 4 | Further comparison of GegSeg with semi-supervised segmentation methods. GenSeg's in-domain generalization performance compared to baseline methods including CTBCT, DCT, and MCF, when used with UNet or DeepLab in segmenting placental vessels, skin lesions, polyps, intraretinal cystoid fluids, foot ulcers, breast cancer, and lungs utilizing the FetReg, FPD, DermQuest, CVC-Clinic, KVASIR, ICFluid, FUSeg, BUID, and COVID datasets. In all panels, bar heights represent the mean, and error bars indicate the standard deviation across three independent runs with different random seeds. Results from individual runs are shown as dot points. Source data are provided as a Source Data file.

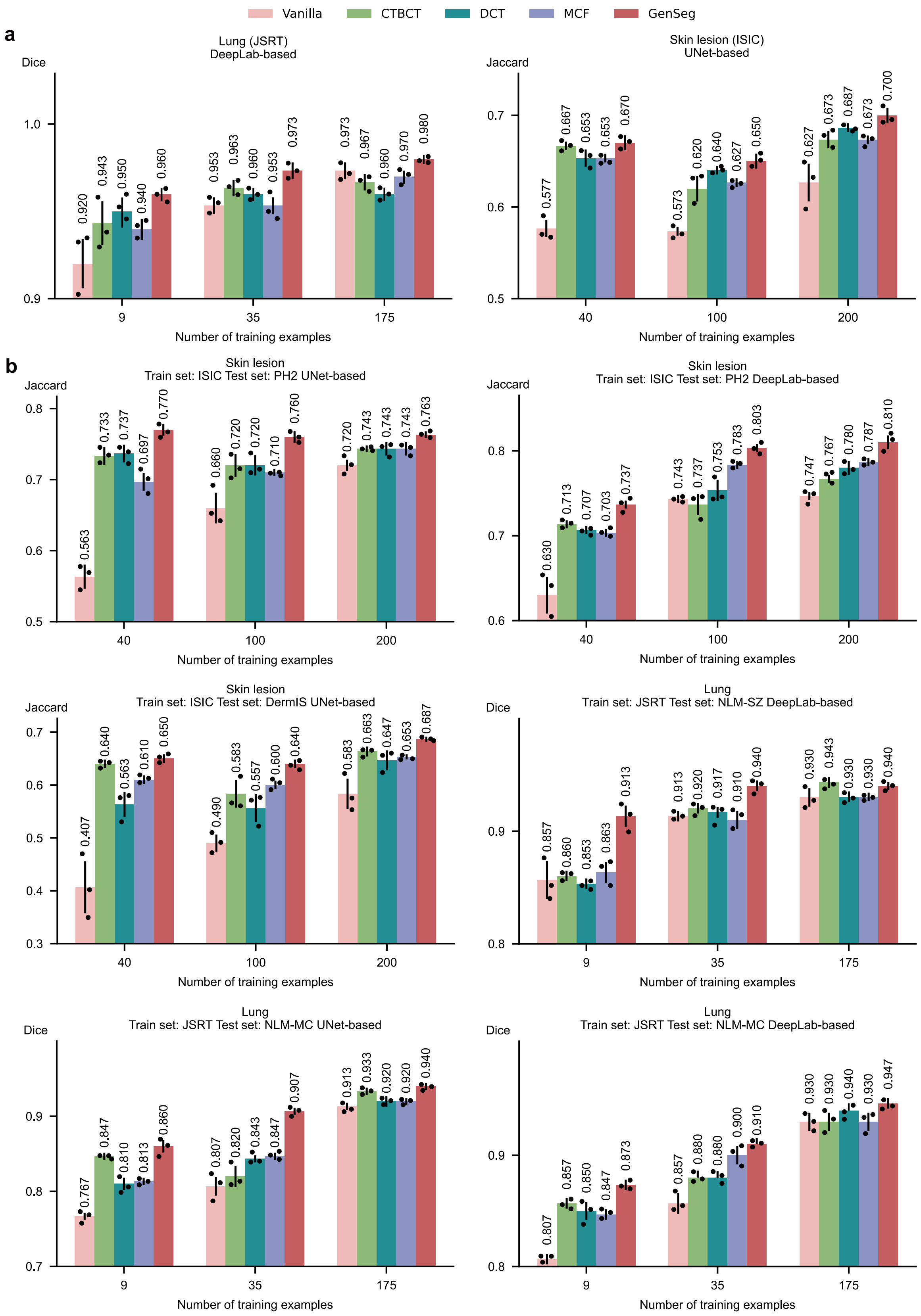

Supplementary Fig. 5 | Further comparison of GegSeg with semi-supervised segmentation methods across varying numbers of training examples. a, Comparison of in-domain generalization performance for segmenting lungs (using the JSRT dataset) and skin lesions (using ISIC). **b**, Comparison of out-of-domain generalization performance for segmenting skin lesions (using ISIC for training, and PH2 and DermIS for testing) and lungs (using JSRT for training, and NLM-SZ and NLM-MC for testing). In all panels, bar heights represent the mean, and error bars indicate the standard deviation across three independent runs with different random seeds. Results from individual runs are shown as dot points. Source data are provided as a Source Data file.

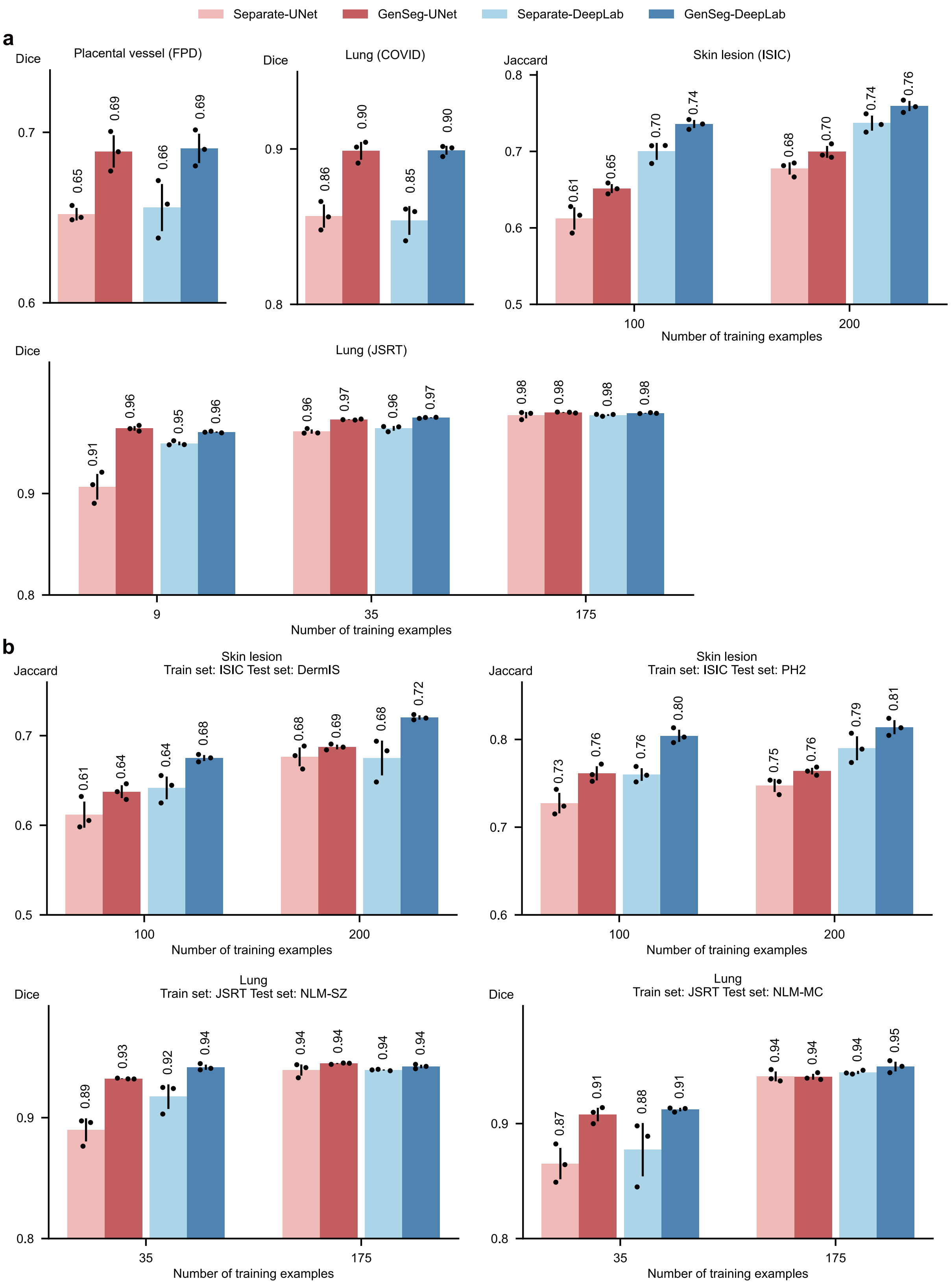

Supplementary Fig. 6 | Further comparison of GenSeg's end-to-end data generation mechanism with baselines' separate generation mechanism. a, GenSeg's end-to-end generation mechanism greatly improves models' in-domain generalization performance, when used UNet and DeepLab in segmenting placental vessels, lung regions, and skin lesions using FPD, COVID, ISIC, and JSRT datasets. **b,** GenSeg's end-to-end generation mechanism greatly improves models' out-of-domain generalization performance, when used UNet and DeepLab in segmenting skin lesions (using ISIC for training, and DermIS and PH2 for testing), and lung regions (using JSRT for training, and NLM-SZ and NLM-MC for testing). In all panels, bar heights represent the mean, and error bars indicate the standard deviation across three independent runs with different random seeds. Results from individual runs are shown as dot points. Source data are provided as a Source Data file.

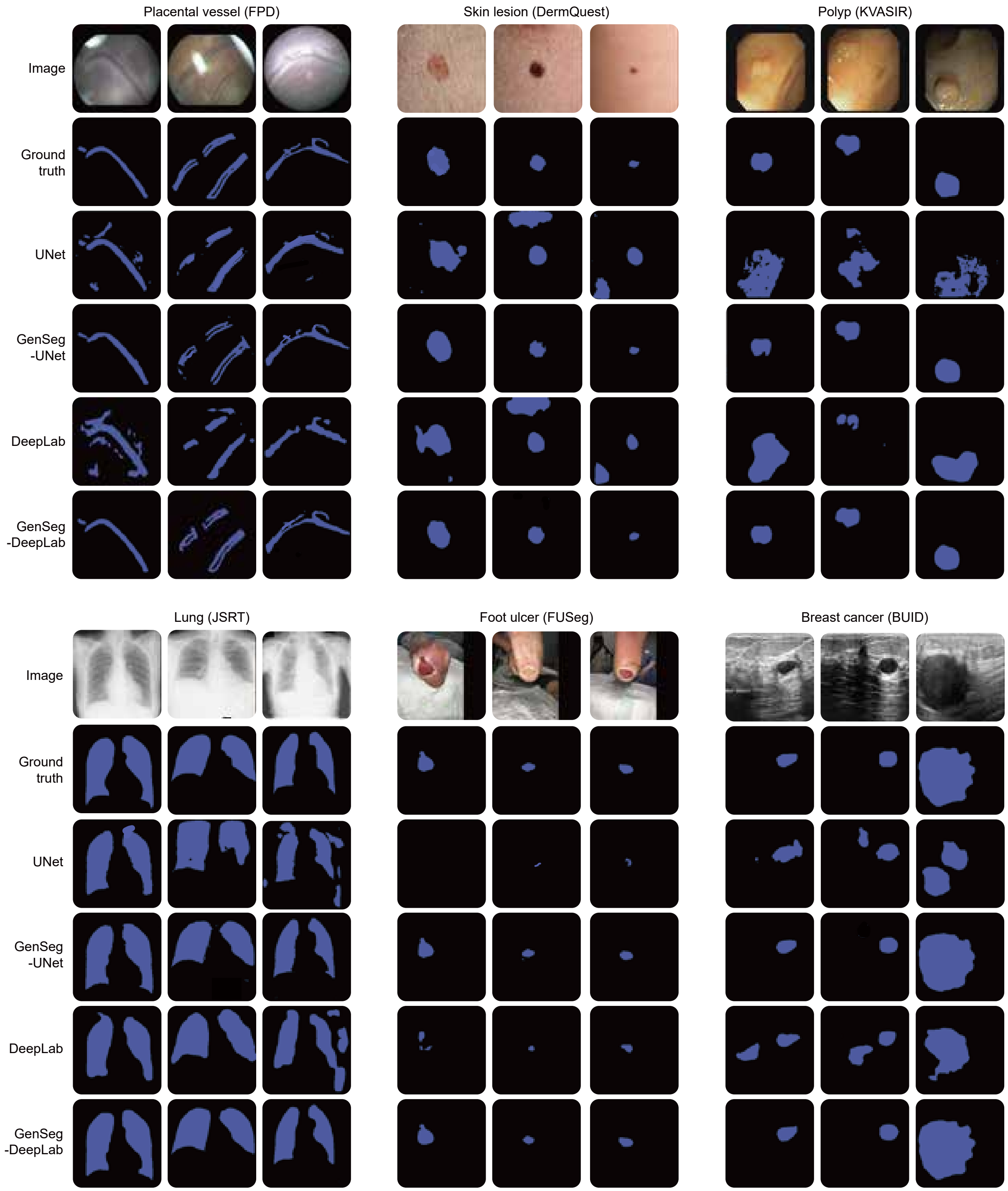

Supplementary Fig. 7 | Additional visualizations of predicted segmentation masks. Visual comparisons of segmentation performance on six medical image segmentation tasks: placental vessel segmentation, skin lesion segmentation, polyp segmentation, lung segmentation, foot ulcer segmentation, and breast cancer segmentation. Each column shows one test sample from the respective dataset and its corresponding qualitative results. All qualitative examples are sourced from publicly available sources.

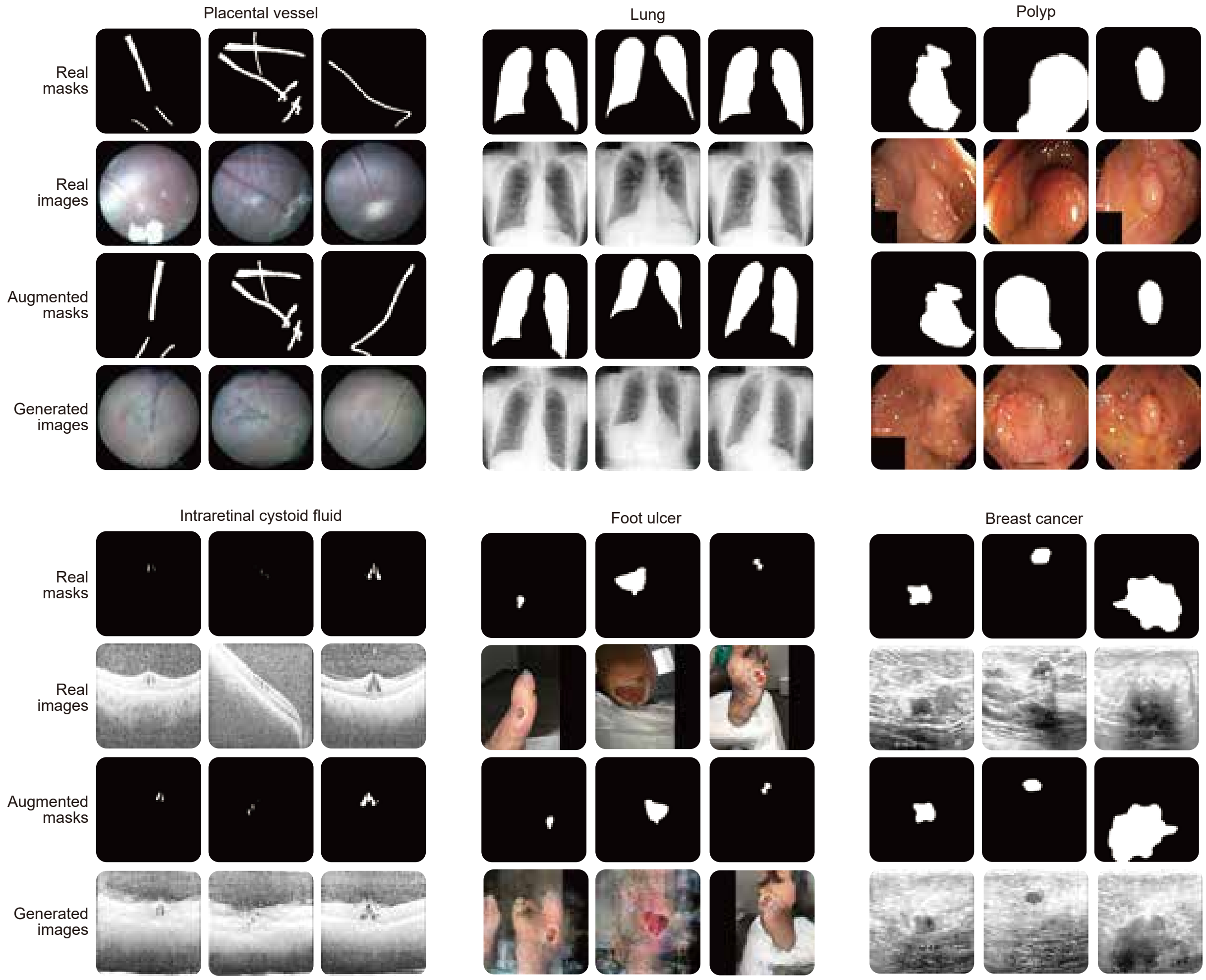

Supplementary Fig. 8 | Visualizations of image-mask pairs generated by GenSeg. Synthetic segmentation masks and medical images generated by GenSeg in tasks of segmenting placental vessels, lungs, polyps, intraretinal cystoid fluid, foot ulcers, and breast cancer. All qualitative examples are sourced from publicly available sources.
